# An NS5A single optimized method to determine genotype, subtype and resistance profiles of Hepatitis C strains

**DOI:** 10.1371/journal.pone.0179562

**Published:** 2017-07-20

**Authors:** Elisabeth Andre-Garnier, Bernard Besse, Audrey Rodallec, Olivier Ribeyrol, Virginie Ferre, Caroline Luco, Laura Le Guen, Nathalie Bourgeois, Jérôme Gournay, Eric Billaud, François Raffi, Marianne Coste-Burel, Berthe-Marie Imbert-Marcille

**Affiliations:** 1 Service de Virologie, CHU Nantes, Nantes, France; 2 Centre de Recherche en Transplantation et Immunologie UMR1064, INSERM, Université de Nantes, Nantes, France; 3 Plateforme de séquençage, CHU Nantes, Nantes, France; 4 Service d’Hépato-gastroentérologie, CHU Nantes, Nantes, France; 5 Service des Maladies Infectieuses et Tropicales, CHU Nantes, Nantes, France; University of North Carolina at Chapel Hill School of Dentistry, UNITED STATES

## Abstract

The objective was to develop a method of HCV genome sequencing that allowed simultaneous genotyping and NS5A inhibitor resistance profiling. In order to validate the use of a unique RT-PCR for genotypes 1–5, 142 plasma samples from patients infected with HCV were analysed. The NS4B-NS5A partial region was successfully amplified and sequenced in all samples. In parallel, partial NS3 sequences were analyzed obtained for genotyping. Phylogenetic analysis showed concordance of genotypes and subtypes with a bootstrap >95% for each type cluster. NS5A resistance mutations were analyzed using the Geno2pheno [hcv] v0.92 tool and compared to the list of known Resistant Associated Substitutions recently published. In conclusion, this tool allows determination of HCV genotypes, subtypes and identification of NS5A resistance mutations. This single method can be used to detect pre-existing resistance mutations in NS5A before treatment and to check the emergence of resistant viruses while undergoing treatment in major HCV genotypes (G1-5) in the EU and the US

## Introduction

Chronic HCV infection, recently re-estimated to affect between 64 and 103 million people worldwide [1] is a major public health problem. Before 2011, standard treatment consisted of Pegylated Interferon (PEGINF) and ribavirin (R). This treatment allowed viral eradication in only 40–50% of patients with genotype 1 (the more frequent genotype) and 80% in HCV genotype 2 (the easiest genotype to treat with this combination) [2].

Increasing knowledge of HCV molecular biology, the establishment of robust HCV replication models and cell culture systems have enabled the development of Direct-Acting Antiviral Agents (DAAs) to treat HCV infection. The expanding armamentarium of DAA combinations for the treatment and cure of HCV have provided great benefits for the infected population with a success rate of up to 90% (Sustained Virologic Response SVR).NS5A inhibitors are one of the four classes of DAAs authorized by the FDA (Food and Drug Administration) and EMA (European Medecines Agency) to treat HCV infection. Other classes are the protease inhibitors (NS3 inhibitors), the nucleotide analogue inhibitors of the RNA-dependent RNA polymerase (RdRp), the NS5B protein and the non nucleosidic NS5B inhibitors [3].

The combination of the high level of HCV replication and the lack of proofreading by the RNA-dependent RNA polymerase leads to a high degree of genetic variability resulting in different genotypes at the population scale [4] and in the constitution of distinct viral quasi-species in each infected individual [5]. Consequently, in this new context of DAA treatment, genetic variability must be taken into account. All the DAA weren’t pangenotypic and the genotype is essential to determine which association of antiviral drugs can be used [6]. When a DAA is administrated, a positive selection of viral variants with a reduced susceptibility to this drug define viral resistance. This is accompanied by the emergence of Resistant Variants (RVs). Another aspect of variability consists in the possibility of baseline Resistance Associated Substitutions (RASs) which have the potential to reduce antiviral therapy efficacy [3]. The specific DAA eligibility, resistance prevalence and efficacy of treatment depend on the HCV geno- and subtypes [6] [7].

To improve the monitoring of HCV infection and determine the baseline, emergence and persistence of RVs, the development of HCV gene target sequencing is required in clinical virology laboratories. In our team, we already have a pangenotypic sequencing technique for NS3 and NS5B genes [8], and therefore we wanted to develop a similar technique for the NS5A gene.

## Material and methods

### Samples

The protocol was evaluated on samples from 142 patients (Nantes University Hospital). These registered HCV patients gave written informed consent before including their samples in a registered biobank. The Local biobank entitled "Infectiologie" was registered by French Relevant Authority (Minsitry in charge of Research) Under ref. DC-2011-1399 and approved by French EC (CPP ouest IV), on November the 8th of 2011.

Blood samples were drawn as part of the patient’s routine follow-up and no additional sample was required. HCV RNA viral loads were performed on the Roche COBAS AmpliPrep/COBAS TaqMan with a lower limit of detection of 15 IU/ml.

Routine genotyping was based on the NS3 sequencing technique developed in our lab [8]. The distribution of HCV genotypes from these clinical samples was: Genotype 1 (G1) n = 61 (42.9%) G2 n = 18 (12.7%) G3 n = 36 (25.4%) G4 n = 23 (16.2%) G5 n = 4 (2.8%) (Table 1). Neither G6 nor G7 were found (Table 1).

**Table 1:** Genotype and subtype distribution of samples.

		Subtype													
Type	% of patients (No)	*	a	b	c	d	e	f	h	i	k	l	q	r	t
**1**	42.9 (61)		29	29			1		1			1			
**2**	12.7 (18)	5	3	2	1					1	4	2			
**3**	25.4 (36)	** **	36												
**4**	16.2 (23)	1	5		2	6		4					1	3	1
**5**	2.8 (4)		4												
**Total:**	100 (142)														

* unassigned subtype

### Design of oligonucleotide primers

In order to amplify the NS4B-NS5A region, different degenerate primers were designed (Table 2). 293 HCV NS4B-NS5A sequences (147 G1 and 146 G2 to G5) extracted from databases (GenBank and the Los Alamos National Laboratory) were aligned with SeqScape software (version 2.5, Applied Biosystems) which enabled the identification of a segment (6066–6882 nucleotides) for the design of consensus primers. We used a unique sense primer NS5A-2F (26 nucleotides including 6 degenerate positions). In order to improve the PCR regardless of the genotypes 1 to 5, two reverse primers were chosen and used together: NS5A-R for G1,G2, G4, G5 and NS5A-R3 for G3, located at the same region. They are used together (25 nucleotides including 8 degenerate positions). To facilitate the sequencing reaction, the M13 universal primers were added to the 5 'end of each PCR primer.

**Table 2:** Primers used for PCR reactions (genotypes 1 to 5).

Primer name	Sense	Sequence primers (5’ to 3’)	Numbering H77
NS5A-2F	F	M13F*-GGIGARGGIGCIGTICARTGGATGAA	6066–6091
NS5A-R	R	M13R*-TRTGRGAIGGRTCIGTIARCATIGA	6882–6858
NS5A-3R	R	M13R-TRTGRGAIGGRTCICTIARCATIGA	6882–6858

*Universal primers: sequences not shown

Y = C or T, R = A or G, V = A or C or G, I = d-Inosine used as “universal” nucleotide replace any nucleotide

### Extraction, amplification and sequencing reaction

Total nucleic acids were extracted from 1 ml of plasma in a 60μl elution volume using the QIAsymphony DSP Virus/Pathogen Kit (catalog N° 937055) with an automated Nucleic acid extractor QIAsymphony SP (QIAgene, Courtaboeuf, France). Reverse transcription and PCR were performed using the PrimeScript One Step RT-PCR kit (ref catalog RR064A TaKaRa, Otsu, Japan) with modifications of the manufacturer's recommendations. The kit was used by running two separate steps: reverse transcription followed by PCR.

RNA was denatured to disrupt strong secondary structures by incubating 10mn at 65°C then placing samples immediately on ice. Reverse transcription was performed in a 15μl RT mix containing 5μl of the linearized RNA, 1X premix buffer, 2.5μM random hexamers (Fermentas, Burlington, Canada), 0.1μg/μl T4 Gene 32 (BioLabs, Ipswich, USA), 0.3μl PrimeScript RT. Thermocycling conditions were as follows: initial random hexamer annealing at 30°C for 5min, reverse transcription at 42°C for 5min, enzyme destruction and initial cDNA denaturation at 95°C for 15s, followed by a 4°C cooling step.

PCR was performed in a 30μl volume containing the 15μl cDNA, 1X premix buffer, the sense primer NS5A-2F at 0.8 μM, and the two antisense primers used together NS5A-R and NS5A-3R at 0.4 μM each (Eurogentec S.A. Herstal, France), 0.05 U/μl TaKaRa Ex Taq TM HS (TaKara, Otsu, Japan) with a proofreading activity achieved by a 3’ -> 5’ exonuclease to ensure a high fidelity performance. The PCR cycling conditions were: 94°C for 3min, followed by 45 cycles of 94°C for 15s, 48°C for 30 sec +0.3°C/cycle, 72°C for 1min, then a 4°C cooling step.

The amplified products were then analyzed by electrophoresis through a 2.2% Agarose gel (FlashGel System Lonza, Rockland, ME USA)

PCR products were purified using the enzymatic method ExoSAP-IT (USB, Cleveland Ohio). Bidirectional sequencing was performed using the fluorescent dye terminator method (Big Dye version 1.1 Cycle Sequencing Kit, Applied Biosystems, Courtaboeuf, France) with the M13 universal forward and reverse primers.

Direct sequencing reaction products were purified through Sephadex G50 columns (GE Healthcare, Little Chalfont, UK,) in a 96 well plate MultiScreen (Merck Millipore, Cork, IRL) Sequencing was performed on an Applied Biosystems ABI 3130 Sequence Analyser.

Nucleotide and amino acid sequence analyses were performed using Seqscape software (version 2.5, Applied Biosystems) to determine genotype, subtype and detect the presence of previously identified substitutions known to confer resistance to NS5A inhibitors (Resistance Associated Substitutions: RASs).

### Phylogenetic analysis

**Fig 1:**
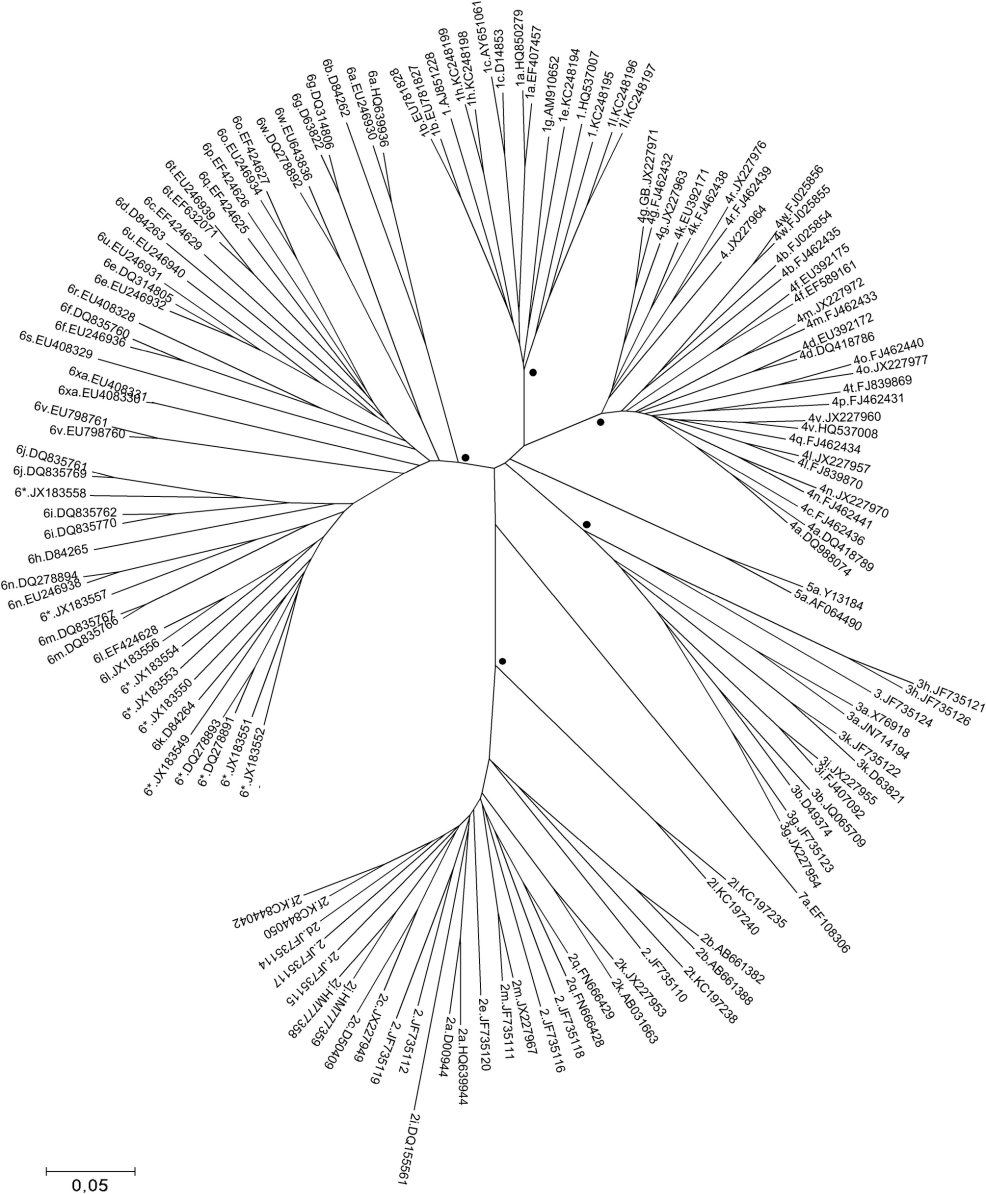
Phylogenetic neighbour joining tree of the HCV NS4B-NS5A fragment sequences (6084–6057 numbering H77) from 141 confirmed HCV genotype/subtype or unassigned reference sequences, identified by accession number. 136 were selected by Smith et Al[4]and five sequences strains were added (subtypes 2l KC197235, KC197240, 2f D49754, D49757 and 2r KC197238). The dot indicates the lowest branch with a bootstrap test >95% (n = 1000 replicates) for each genotype (1,2,3,4,6) cluster. The evolutionary distances were computed using the Maximum Composite Likelihood method (MEGA5).There were a total of 765 positions in the final dataset.

**Fig 2:**
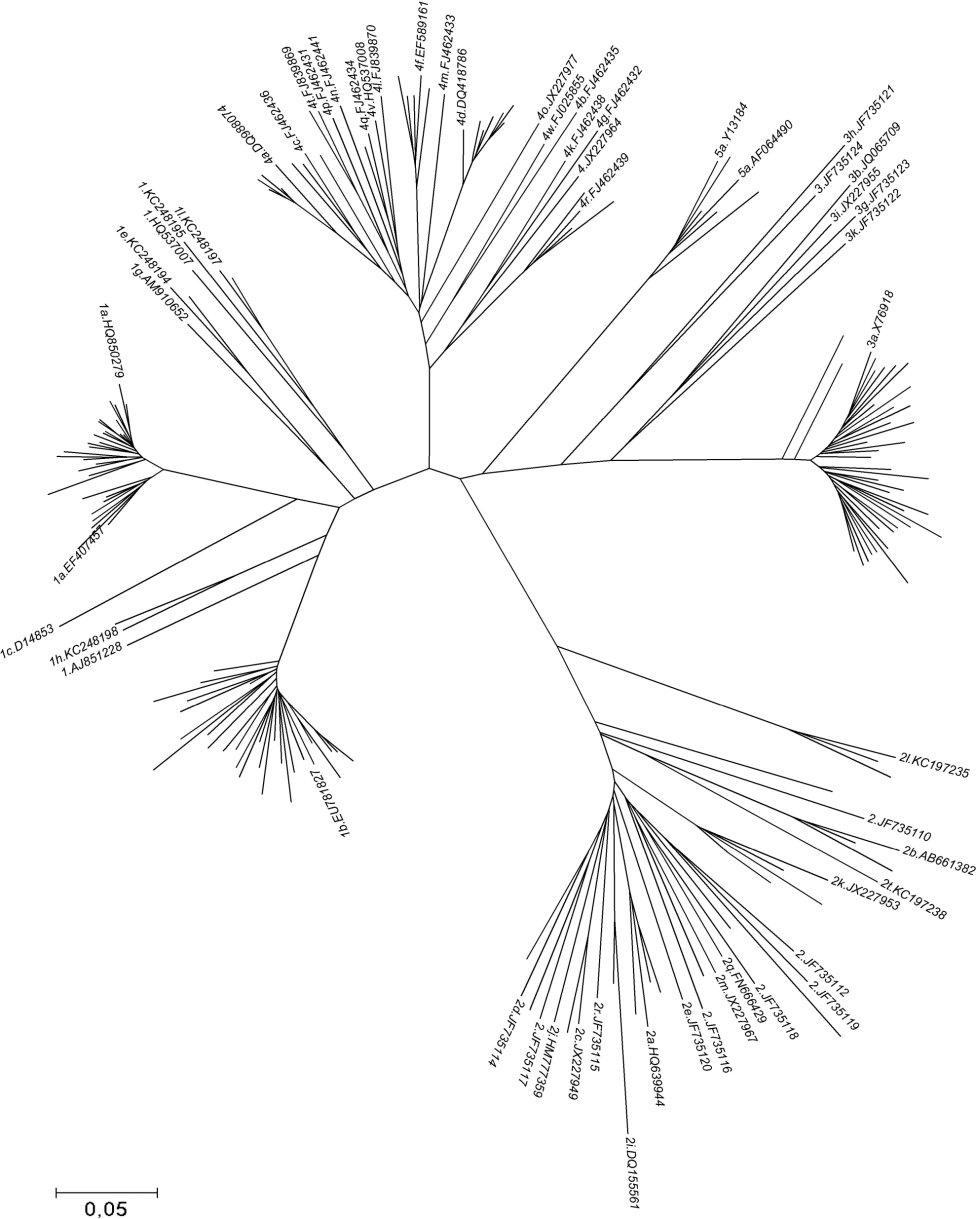
Phylogenetic tree of the 142 clinical sequences NS4B-NS5A from patients (taxon name not shown) aligned with HCV references sequences (accession number shown). To lighten the phylogenetic tree, only genotype 1,2,3,4,5a and one reference sequence per subtype were used to build the neighbour joining tree. Genetic distances were calculated using the Maximum Composite Likelihood method (MEGA5).

To investigate the suitability of the nucleotide NS4B-NS5A sequence fragment for genotyping, NS4B-NS5A fragments from complete reference genomes provided in Smith et al [4] were aligned and a phylogenetic analysis was performed using the Neighbor-Joining method (MEGA5 software, [9]) The reliability of the phylogenetic clustering was evaluated using bootstrap analysis with 1000 replicates (Fig 1). The 142 sequences from the clinical samples were aligned with the same confirmed sequences and a phylogenetic tree was built using identical parameters (Fig 2). The type and subtype were compared to those obtained by sequencing the NS3 fragment.

### NS5A resistance analysis

Clinical strains (n = 142) were analyzed using geno2pheno [hcv]V0.92 (update April 2016) developed by Max-Planck-Institute for informatics [10]completed with the recently published list of known RASs [11,12].

## Results

### Design of oligonucleotides

Two criteria guided our choice to ensure the specificity and effectiveness of the reaction: the primers should be long enough and the degenerate nucleotide positions need to be balanced along the sequence. These design criteria ensured the efficiency and specificity of the amplification of the expected region of NS5A (AA 1–200). Using inosine instead of “N” (any) nucleotides during the synthesis of primers contribute to ensure efficient hybridization of the primer by reducing the number of different variant primers inside the mixture.

Using M13 universal primers for the sequencing reaction rather than forward and reverse HCV degenerate primers, improves the Sanger sequencing step. The annealing temperature (50°) used in this standard sequencing reaction is optimal for these primers.

### RT-PCR performance

This unusual protocol using a one-step RT-PCR kit in two steps was described in a previous publication [8]. It has shown its efficacy for the amplification of an NS3 pangenotypic fragment from samples (to date 0.9% failed, n = 1422). This protocol was successfully transposed to the new NS5A amplification. All the genotypes and subtypes isolated from clinical samples were amplified without the use of nested-PCR. Sensitivity was not evaluated but the mean of the viral load was 6.2 log UI/ml in our cohort. The lowest sample viral load (2.8 log UI/ml) was successfully amplified.

This protocol has also been applied to samples provided by an external laboratory for an independent International External Quality Assessment (EQA): Quality Control for Molecular Diagnostics (QCMD 2016 ref N° QAV34117_1). The genotypes and subtypes were correctly identified for all 8 samples.

### Phylogenetic analysis results

The comparison of the phylogenetic trees obtained with complete confirmed HCV sequences realized by Smith et al [4] and those built with the NS4B-NS5A fragment of these sequences showed an identical distribution of the genotypes and subtypes (Fig 1). The bootstraps calculated were up to 95% for each genotype cluster. Genotyping and subtyping were possible due to the high percentage of substitutions between genotypes (mean: 42,3%, values are detailed in Table 3) and within genotypes (mean G1 26.6% (24.7 to 31.5), G2 25.6% (19 to 39.5), G3 31.6% (25 to 41.3), G4 22% (14 to 28.2) G6 32.9% (18.1 to 44.3).

**Table 3:** Divergence over sequence pairs between HCV genotypes using nucleotides sequences of the reference strains (%).

Genotype	1	2	3	4	5	6
**2**	49					
**3**	44.2	55.9				
**4**	36	49.4	43.5			
**5**	39	48.7	42.7	36		
**6**	42.4	52.8	46.6	41.1	41.3	
**7**	49	55	59.1	49.2	49.8	53

The percentage of substitutions from averaging over all sequence pairs between genotypes are shown. Analyses were conducted using the Maximum Composite Likelihood model.The analysis involved 129 nucleotide sequences. There were a total of 765 positions in the final dataset.

The 142 NS4B-NS5A sequences from the clinical samples were aligned with the same confirmed sequences and a phylogenetic tree was built to identify genotypes and subtypes using the same parameters (Fig 2). In parallel, all samples were successfully amplified and sequenced with the NS3 protocol. All genotypes and subtypes obtained in both protocols were 100% identical.

### Identification of pre-existing RASs (Table 4)

In these clinical samples, 18% harboured pre-existing RASs and there was no difference between the different genotypes evaluated in terms of RAS prevalence. Moreover substitutions conferring a >100 fold increase NS5A inhibitor EC50 *in vitro*, like Q30H/R alone or associated with L31M, Y93H, were present in our cohort with a prevalence of 7%.

**Table 4:** Natural prevalence of NS5A inhibitor RAS revealed in the 142 sequenced samples.

	Associated with genotype or subtype							
	1a n = 29	1b n = 29	2 n = 18	3 n = 36	4 n = 23		5 n = 4	
K24G/N/R	no							
T24A			no					
K26E	no							
M28A/G/T/S/V	no							
L28M/T		No						
L/F/28/M/V/S			no					
L28V					n = 3 L28V			
L28I							no	
M28T				no				
P29S		No						
Q30C/D/E/G/H/I/L/K/R/S/T/Y	n = 1 Q30R+L31M							
R30G/H/P/Q		n = 1 R30Q n = 1 R30Q+L31M						
L30H/S			no					
A30K				n = 2 A30K				
L30H					no			
L31I/F/M/V	n = 1 Q30R+L31M	n = 3 L31M n = 1 R30Q+L31M						
L31M/V			n = 12 L31M	no				
L31V							no	
P32L/S	no	no						
S38F	no							
H58D/L/R	no							
P58D/S		no						
E62D		no						
A92K/T	no	n = 1 A92K						
Y93C/F/H/L/N/R/S/T/W	no							
Y93C/H/N/S		n = 1 Y93H						
Y93H			no	no				
Y93H/R					no			
**Total sequences + RASs :**	**1**	**7**	**12**	**2**	**3**		**0**	

no: lack of known RASs in our cohort, grey cells: RASs associated with genotype or subtype

## Discussion

HCV DAA therapy represents a revolution in HCV treatment [13,14].Today, some DAAs do not have a pangenotypic action and experts define different combinations according to HCV genotype. Thus, HCV genotype determination is always recommended before starting a new DAA treatment. Resistance of hepatitis C to antiviral treatment is a new challenge for hepatologists and is associated with different questions to which virologists could respond. First in a real-life clinical setting, what is the impact of pre-existing RASs in the success of therapy [15]? Secondly, even if the event is rare [16], after a failed NS5A inhibitor treatment, how long do resistant variants persist, and what is their impact on rapid retreatment? In the event of virologic failure, all experts (AASLD The American Association for Studies of Liver Disease, EASL European Association for the Study of the Liver) recognize the importance of checking the disappearance of resistant variants before re-treating with the same therapeutic class [6]. For all these reasons, we need to develop a robust, easy genotyping and resistance profiling method especially for the NS5A region.

By performing reverse transcription with random hexamers, all HCV RNA types and subtypes are transcribed without any primer selection, as in a one-step protocol. The buffer is optimized for reverse transcription and PCR. Thus it is possible to carry out both reactions separately. Using the entire volume of the reverse transcription reaction to realize the PCR. allows to increase the sensitivity of the amplification. The use of separate kits does not allow the two reactions to be mixed with an equal volume due to the different composition of the reaction buffers [8].

Regarding the feasibility of the approach, the phylogenetic analysis of the NS4B-NS5A HCV sequences is perfectly correlated with that using complete virus coding sequences, as performed by Smith et al [4]. Thus our proposed protocol could be used to determine both genotype/subtype and NS5A polymorphism for any new HCV infected patient. Indeed, the NS4B-NS5A region has recently been described as a candidate to perform genotyping and subtyping for HCV [17].

Although the phylogenetic analysis of the NS4B-NS5A amplified fragment makes it possible to consider the validation of the typing / subtyping of genotypes 6 and 7, we have not validated the amplification of these genotypes. Indeed their prevalence is very low in our country and we have only a few strains in our biobank. Combined with the fact that there is a large number of identified subtypes 6 24 from “a” to “w”), we have not been able to undertake the validation of amplification on this genotype as well as the G7.

On 142 clinical samples the method described herein of NS5A gene amplification and sequencing has shown a perfect match with our routine genotyping method using the NS3 region [8].

This unique NS5A analysis is neither costly nor time-consuming. Indeed the usual protocols first require genotyping [18] and then in a second step, a specific type of RT-PCR to amplify and sequence the target NS5A fragment. Provided our protocol, described here in this study is strictly followed it is easily transposable to other laboratories.

The PCR we used amplifies a fragment of NS5A (domain I) covering amino acids 1 to 200 which contain the reported Resistance Associated Substitutions (RASs) associated with viral failure under daclatasvir, ledispasvir, ombitasvir or velpatasvir NS5A inhibitors. Today, there is no simple and consensual tool to easily interpret the presence of RASs at treatment baseline or in case of virological failure. The advantage of using the Geno2Pheno tool is the regular updating of the database (last updated February 2017) [10]with the results of all publications relating to the resistance of HCV, both *in vitro* and *in vivo*. The list of mutations reported in recent previously cited reviews are interesting because they are described in the phase II and III studies and so are closer to the results of the real clinical practice [10,12].

RASs are often detected at baseline in DAA-naïve patients. In our study, the rate of mutated variants NS5A inhibitors was superior to 15% in agreement with the literature. Indeed in previous studies which reported the proportion of detectable RASs at treatment baseline in various patient populations, the rate of pre-existing RASs is 13% of cases in North America, 14% in Europe, 7% in Asia Pacific and 16% in Oceania. These reports are generally based on population Sanger sequencing which allows detection of mutations with sensitivity between 15% to 20%. Deep sequencing techniques are more sensitive with detection of viral population variants among quasi-species under 15%. However there is a consensus that the cut off reached with the Sanger method is sufficient and relevant in clinical studies [19].

In the literature, most of the data on the impact of natural variants concerns HCV genotype 1. Thus, with sofosbuvir/ledipasvir treatment, the presence of NS5A RASs (conferring a high resistance level to ledipasvir: >100-fold increase in EC50) at baseline affects the chances for a Sustained Virological Response in certain groups of patients. In particular, this is significant in treatment for experienced patients treated for 12 weeks without ribavirin and infected with genotype 1a [20]. The addition of ribavirin prevented the effects of pre-existing NS5A RASs on SVR [21,22]. With therapy combined in a single sofosbuvir/velpastasvir pill, NS5A RASs in patients infected with HCV genotype 3 strain or with decompensated cirrhosis, the impact of baseline RASs can be countered by extending therapy to 24 weeks [23,24]. Few data are available for other genotypes [20]. Real life studies are needed to determine the effect of these pre-existing mutations on the success of the anti HCV treatment and on how to proceed in this context. Do we have to add Ribavirin and/or extend the duration of the therapy?

In the case of virological failure, analyzing RASs is more important with NS5A inhibitors than with NS3 inhibitors because the resistant variants are naturally fitter and therefore persist for years after DAA therapy [25].

With the protocol developed in this study, virologists and hepatologists have a solution to homogenize the response of RVs.

Our method will allow the investigation of pre-existing NS5A RASs which will help to prospectively evaluate their effects. To our knowledge, this is the first method using only one reaction mixture to amplify all main genotypes and subtypes HCV for NS5A. This transposable technique will allow a standardization of NS5A genotyping that could be used to develop a fully automated method for HCV genotype/resistance typing.
